# TMEM2 inhibits hepatitis B virus infection in HepG2 and HepG2.2.15 cells by activating the JAK–STAT signaling pathway

**DOI:** 10.1038/cddis.2016.146

**Published:** 2016-06-02

**Authors:** X Zhu, C Xie, Y-m Li, Z-l Huang, Q-y Zhao, Z-x Hu, P-p Wang, Y-r Gu, Z-l Gao, L Peng

**Affiliations:** 1 Department of Infectious Diseases, Third Affiliated Hospital of Sun Yat-sen University, 600# Tianhe Road, Guangzhou, Guangdong Province, China; 2 Guangdong Provincial Key Laboratory of Liver Diseases, Third Affiliated Hospital of Sun Yat-sen University, Guangzhou, Guangdong Province, China; 3 Key Laboratory of Tropical Disease Control, Ministry of Education, Sun Yat-sen University, Guangzhou, Guangdong Province, China; 4 Department of Traditional Chinese Medicine, Third Affiliated Hospital of Sun Yat-sen University, 600# Tianhe Road, Guangzhou, Guangdong Province, China

## Abstract

We have previously observed the downregulation of TMEM2 in the liver tissue of patients with chronic hepatitis B virus (HBV) infection and in HepG2.2.15 cells with HBV genomic DNA. In the present study, we investigated the role and mechanism of TMEM2 in HepG2 and HepG2.2.15 during HBV infection HepG2 and HepG2.2.15. HepG2 shTMEM2 cells with stable TMEM2 knockdown and HepG2 TMEM2 and HepG2.2.15 TMEM2 cells with stable TMEM2 overexpression were established using lentivirus vectors. We observed reduced expression of TMEM2 in HBV-infected liver tissues and HepG2.2.15 cells. HBsAg, HBcAg, HBV DNA, and HBV cccDNA levels were significantly increased in HepG2 shTMEM2 cells but decreased in HepG2 TMEM2 and HepG2.2.15 TMEM2 cells compared with naive HepG2 cells. On the basis of the western blotting results, the JAK–STAT signaling pathway was inhibited in HepG2 shTMEM2 cells but activated in HepG2 TMEM2 and HepG2.2.15 TMEM2 cells. In addition, reduced and increased expression of the antiviral proteins MxA and OAS1 was observed in TMEM2-silenced cells (HepG2 shTMEM2 cells) and TMEM2-overexpressing cells (HepG2 TMEM2 and HepG2.2.15 TMEM2 cells), respectively. The expression of Interferon regulatory factor 9 (IRF9) was not affected by TMEM2. However, we found that overexpression and knockdown of TMEM2, respectively, promoted and inhibited importation of IRF9 into nuclei. The luciferase reporter assay showed that IRF9 nuclear translocation affected interferon-stimulated response element activities. In addition, the inhibitory effects of TMEM2 on HBV infection in HepG2 shTMEM2 cells was significantly enhanced by pre-treatment with interferon but significantly inhibited in HepG2.2.15 TMEM2 cells by pre-treatment with JAK1 inhibitor. TMEM2 inhibits HBV infection in HepG2 and HepG2.2.15 by activating the JAK–STAT signaling pathway.

Chronic hepatitis B virus (HBV) infection is a global public health challenge. It has been estimated that two billion people worldwide are infected with HBV^1^ and that ~350 million people have chronic HBV infection, which is associated with cirrhosis, liver failure, and hepatocellular carcinoma. Up to one million deaths annually are caused by HBV-related diseases.^2^ However, the mechanism by which HBV infects HepG2 and HepG2.2.15 cells is not fully understood. Our previous study investigating susceptibility to HBV revealed significant differences in the expression of the p.Ser1254Asn gene between healthy individuals and patients with HBV infection. Expression of the transmembrane protein TMEM2 encoded by p.Ser1254Asn in normal liver tissues and HepG2 cells was significantly higher than that in liver tissues of patients with chronic HBV infection and in HepG2.2.15 cells with HBV genomic DNA, respectively,^3^ suggesting that TMEM2 has an important role in inhibiting HBV infection in HepG2 and HepG2.2.15 cells. TMEM2 belongs to the interferon-inducible transmembrane protein superfamily. The biological functions of TMEM2 remain largely unknown. It has been reported that other members of the interferon-inducible transmembrane protein superfamily exhibit interferon-mediated antiviral functions.^4, 5^ The JAK–STAT signaling pathway regulates cell growth, survival, and differentiation, and is involved in pathogen resistance. Interestingly, Li *et al.*^6^ reported that inhibition of STAT1 methylation led to the resistance of HBV to interferon alpha treatments. On the basis of previous findings, we hypothesized that the JAK–STAT signaling pathway is involved in the anti-HBV activity of TMEM2. In the present study, we investigated the interactions between TMEM2 and the JAK–STAT signaling pathway during HBV infection in HepG2 and HepG2.2.15 cells. First, we performed gain- and loss-of-function assays in HepG2 and HepG2.2.15 cells by stable TMEM2 overexpression or silencing. Second, the expression of a number of key components of the JAK–STAT signaling pathway was evaluated, and the effects of the JAK–STAT signaling pathway on TMEM2-mediated anti-HBV activities were investigated.

## Results and Discussion

### Expression of TMEM2 in liver tissues and HepG2 and HepG2.2.15 cells

**Figure 1:**
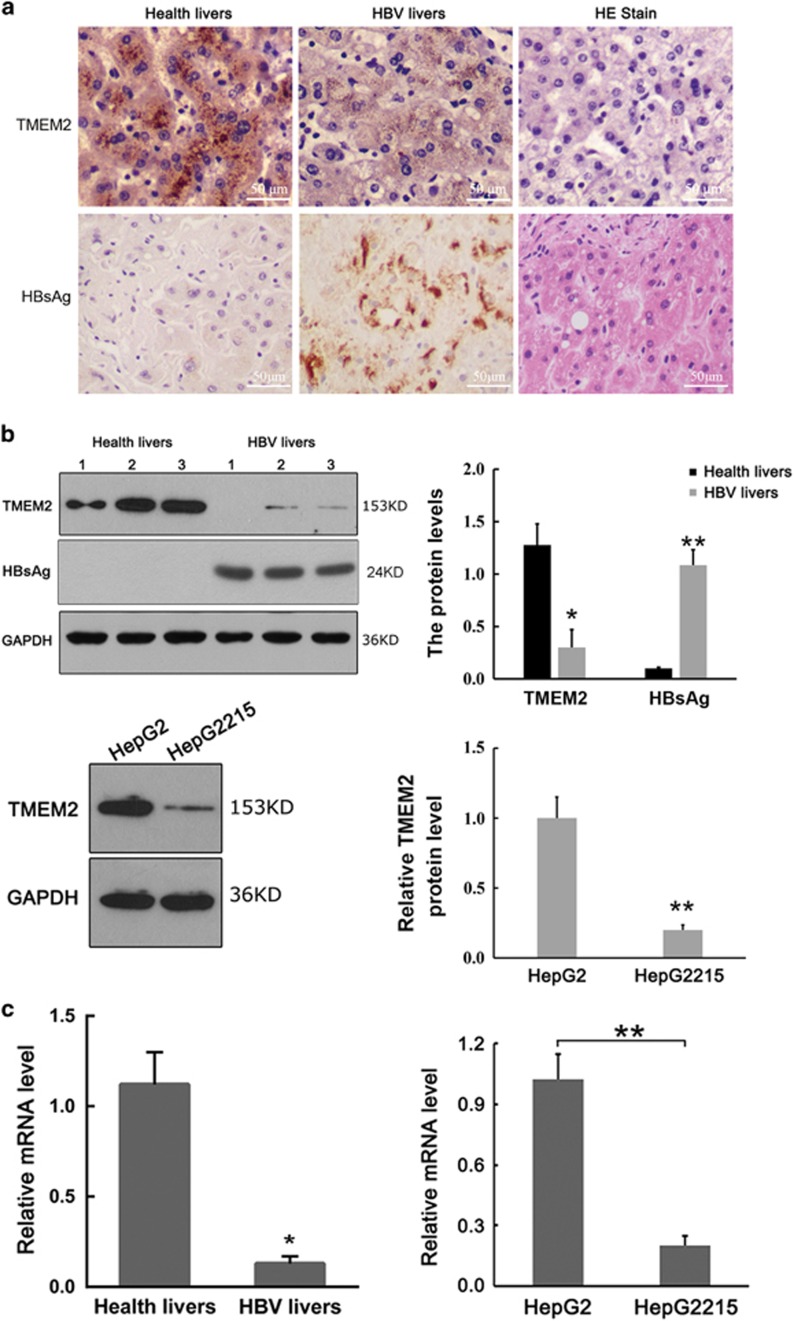
Expression of TMEM2 in liver tissues and HepG2 and HepG2.2.15 cells. (**a**) Expression of TMEM2 in liver tissues based on IHC. (Left) Grainy staining of TMEM2 was observed in the cytoplasm in normal liver tissues. (Middle) Reduced TMEM2 expression in the liver tissues from a patient with chronic HBV infection. (Right) HE staining of normal liver tissues (scale bar, 50 *μ*m). (**b**) Western blot results showed reduced expression of TMEM2 in the liver tissues from a patient with chronic HBV infection compared with normal liver tissues (above) (healthy livers compared with HBV livers: *P<0.05, **P<0.01). Western blot results showed reduced expression of TMEM2 in HepG2.2.15 cells compared with HepG2 cells (below) (HepG2 *versus* HepG2215: **P<0.01). (**c**) RT-qPCR results showed reduced TMEM2 mRNA levels in the liver tissues from a patient with chronic HBV infection compared with that in normal liver tissues (left) (healthy livers *versus* HBV livers: *P<0.05). RT-qPCR results showed reduced TMEM2 mRNA levels in HepG2.2.15 cells compared with HepG2 cells (right) (HepG2 *versus* HepG2215: **P<0.01). Error bars are presented as S.D.

IHC demonstrated strong expression of TMEM2 in the cytoplasm in normal liver tissue and significantly reduced expression of TMEM2 in liver tissues from patients with chronic HBV infection (Figure 1a). The western blot analysis also showed a significant reduction of TMEM2 expression in liver tissues from patients with chronic HBV infection and HepG2.2.15 cells with HBV genomic DNA compared with normal liver tissues and HepG2 cells without HBV genomic DNA, respectively (Figure 1b). In addition, the mRNA level of TMEM2 in liver tissues from patients with chronic HBV infection and HepG2.2.15 cells with HBV genomic DNA was significantly lower than that in normal liver tissues and HepG2 cells without HBV genomic DNA, respectively (Figure 1c; *P<0.05, **P<0.01).

### Successful establishment of stable TMEM2-overexpressing or silenced cell lines

**Figure 2:**
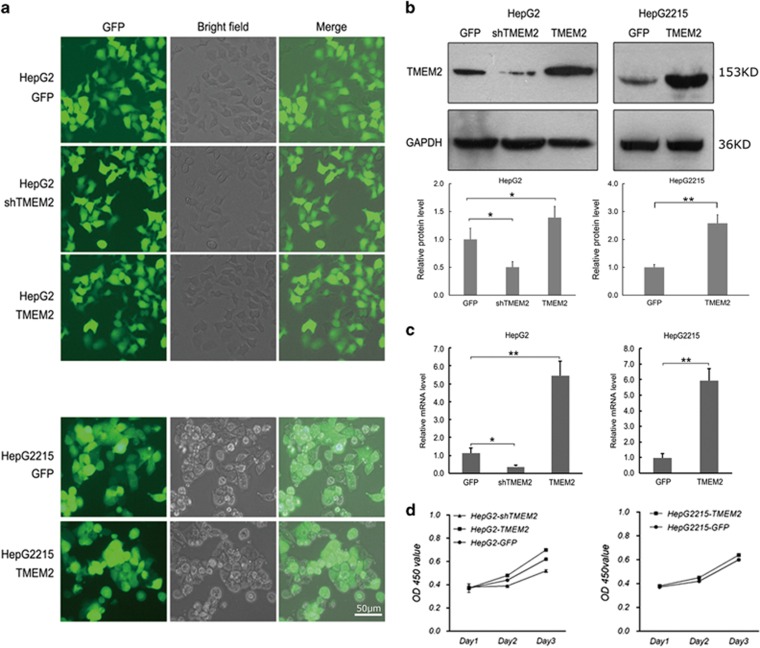
Expression of TMEM2 in stable cell lines. (**a**) HepG2 GFP cells (the control HepG2 cells), HepG2 shTMEM2 cells with TMEM2 silenced, HepG2 TMEM2 cells with TMEM2 overexpression, HepG2.2.15 GFP cells (normal HepG2.2.15 cells), and HepG2.2.15 TMEM2 cells with TMEM2 overexpression. (**b**) Western blot results showed reduced expression of TMEM2 in HepG2 shTMEM2 cells (GFP *versus* HepG2 shTMEM2: *P<0.05; GFP *versus* HepG2 TMEM2: *P<0.05) and increased expression of TMEM2 in HepG2 TMEM2 and HepG2.2.15 TMEM2 cells (GFP *versus* HepG2215 TMEM2: **P<0.01). (**c**) RT-qPCR results showed reduced levels of TMEM2 mRNA in HepG2 shTMEM2 cells (GFP *versus* HepG2 shTMEM2: *P<0.05; GFP *versus* HepG2 TMEM2: **P<0.01) and increased mRNA levels of TMEM2 in HepG2 TMEM2 and HepG2.2.15 TMEM2 cells (GFP *versus* HepG2215 TMEM2: **P<0.01)). (**d**) Effect of TMEM2 silencing or overexpression on the kinetics of cell growth/cell death. Overexpression of TMEM2 slightly promoted the proliferation of HepG2 and HepG2.2.15 (*P*>0.05), and TMEM2 silencing slightly repressed the proliferation of HepG2 cells (*P*>0.05). Error bars are presented as S.D.

Lentiviral vectors were used to establish stable TMEM2-overexpressing (HepG2 TMEM2 and HepG2.2.15 TMEM2 cells) or silenced (HepG2 shTMEM2 cells) cell lines (Figure 2a). Western blot and real-time quantitative PCR (RT-qPCR) results demonstrated significantly increased expression of TMEM2 in HepG2 TMEM2 and HepG2.2.15 TMEM2 cells compared with HepG2 and HepG2.2.15 cells (*P*<0.01), respectively, and significantly reduced expression of TMEM2 in HepG2 shTMEM2 cells compared with HepG2 cells (Figures 2b and c; *P*<0.05). The effect of TMEM2 silencing or overexpression on the kinetics of cell growth/cell death was determined using the MTT assay, as shown in Figure 2d. Overexpression of TMEM2 promoted a slight increase in the proliferation of HepG2 and HepG2.2.15, whereas TMEM2 silencing slightly repressed the proliferation of HepG2 cells.

### TMEM2 inhibited HBV infection in HepG2 and HepG2.2.15 cells

**Figure 3:**
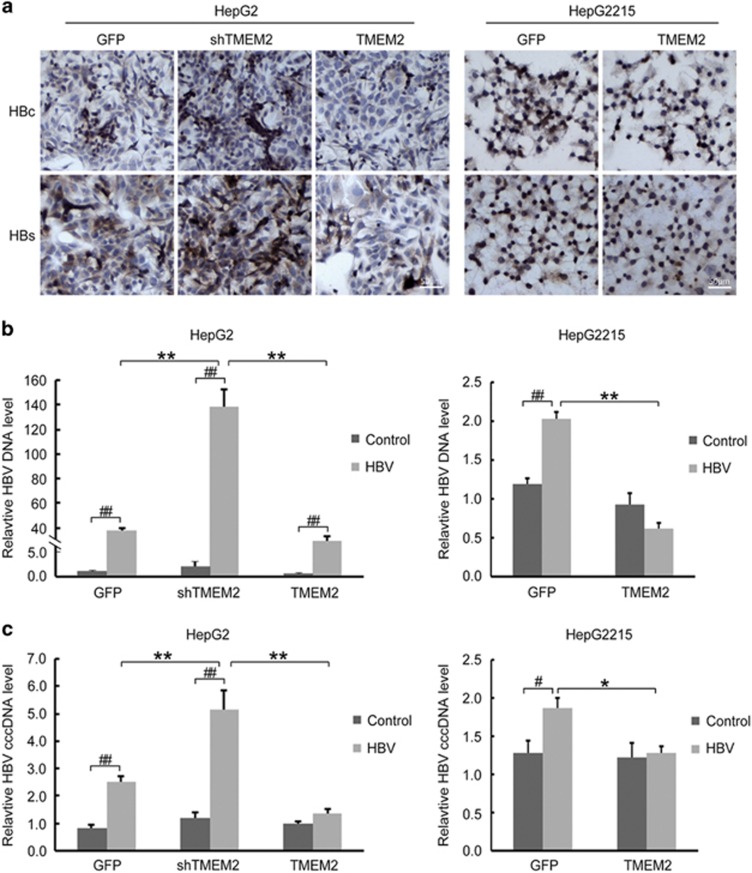
Levels of HBsAg, HBcAg, HBV DNA, and HBV cccDNA in HBV-infected HepG2 and HepG2.2.15 with TMEM2 overexpression or silencing. (**a**) IHC results showed higher levels of HBsAg and HBcAg in HepG2 shTMEM2 cells than in HepG2 GFP cells. The levels of HBsAg and HBcAg were lower in HepG2 TMEM2 cells than in HepG2 GFP cells. In addition, the levels of HBsAg and HBcAg were lower in HepG2.2.15 TMEM2 cells than in HepG2.2.15 GFP cells. HBc: HBcAg; HBs: HBsAg. (**b**) Quantitative analysis of HBV DNA in HBV-infected HepG2 and HepG2.2.15 with TMEM2 overexpression or silencing. After HBV infection, the HBV DNA level increased in all cells. After HBV infection, the level of HBV DNA in HepG2 shTMEM2 cells was higher than that in HepG2 GFP and HepG2 TMEM2 cells (left, **P<0.01; ^##^*P*<0.01). After HBV infection, the level of HBV DNA in HepG2.2.15 GFP cells was significantly increased (right, ^##^*P*<0.01). After HBV infection, the level of HBV DNA in HepG2.2.15 TMEM2 cells was lower than that in HepG2.2.15 GFP cells (right, **P<0.01). (**c**) Quantitative analysis of HBV cccDNA in HBV-infected HepG2 and HepG2.2.15 with TMEM2 overexpression or silencing. After HBV infection, HBV cccDNA levels were increased in HepG2 GFP and HepG2 shTMEM2 cells (left, ^##^*P*<0.01). After HBV infection, HBV cccDNA levels were increased in HepG2 shTMEM2 cells compared with HepG2 GFP and HepG2 TMEM2 cells (left, ^**^P<0.01). After HBV infection, HBV cccDNA were significantly increased in HepG2.2.15 GFP cells (right, ^#^*P*<0.05). After HBV infection, HBV cccDNA levels were lower in HepG2.2.15 TMEM2 cells compared with HepG2.2.15 GFP cells (right, *P<0.05). Error bars are presented as S.D.

HepG2 and HepG2.2.15 cells were cultured in l-DMEM (Dulbecco's modified Eagle's medium) medium containing 10% serum from patients with chronic HBV infection for 48 h. The IHC results demonstrated that the levels of HBsAg and HBcAg in HepG2 shTMEM2 cells were higher than those in HepG2 cells, and the levels of HBsAg and HBcAg in HepG2 TMEM2 and HepG2.2.15 TMEM2 cells were lower than those in HepG2 and HepG2.2.15 TMEM2 cells, respectively (Figure 3a).

The RT-qPCR results demonstrated that the level of HBV DNA in all cells (HepG2, HepG2 shTMEM2, HepG2 TMEM2, and Hep2.2.15), excluding HepG2.2.15 TMEM2 cells, was significantly increased in response to HBV infection (Figure 3b, *P*<0.01). The HBV DNA level in HepG2 shTMEM2 cells was significantly higher than that in HepG2 and HepG2 TMEM2 cells (Figure 3b, left panel, *P*<0.01). In HepG2.2.15 TMEM2 cells, the HBV DNA level was significantly lower than that in HepG2.2.15 cells (Figure 3b, right panel, *P*<0.01). The HBV cccDNA levels in HepG2, HepG2 shTMEM2, and HepG2.2.15 cells were significantly increased in response to HBV infection (Figure 3c). In addition, the HBV cccDNA level in HepG2 shTMEM2 cells was significantly increased compared with that in HepG2 and HepG2 TMEM2 cells (Figure 3c, left panel, *P*<0.01). The HBV cccDNA level in HepG2.2.15 TMEM2 cells was significantly lower than that in HepG2.2.15 cells (Figure 3c, right panel, *P*<0.05).

### Effects of TMEM2 on the JAK–STAT signaling pathway in HBV-infected HepG2 and HepG2.2.15 cells

**Figure 4:**
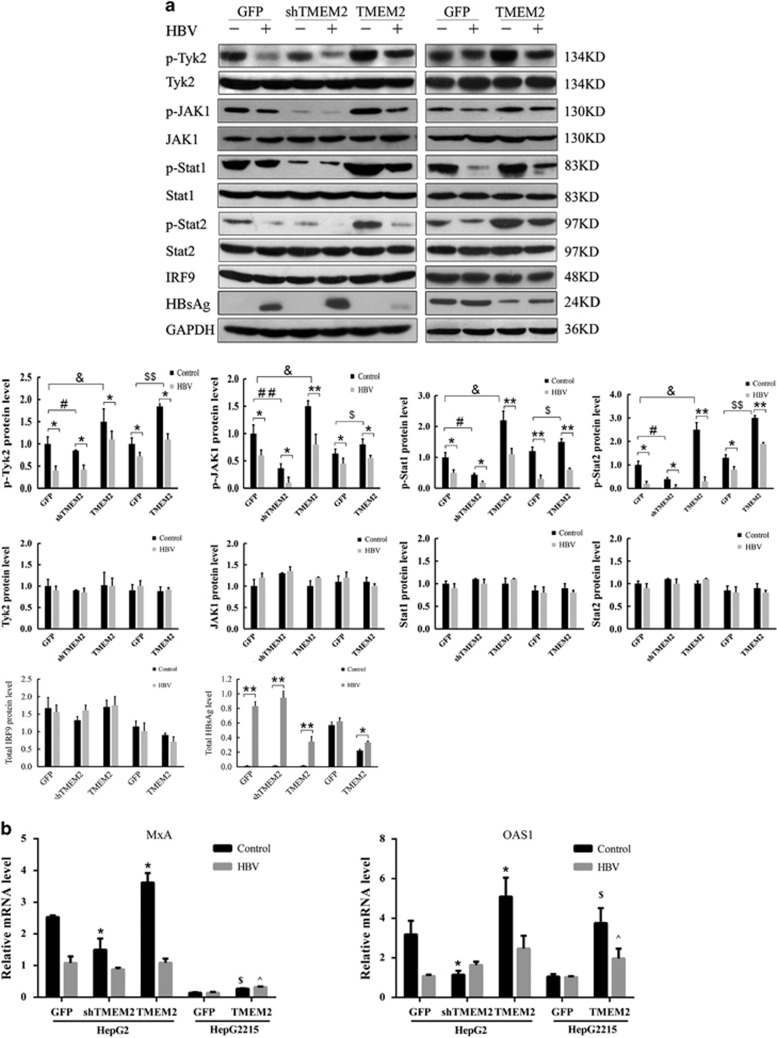
TMEM2 regulated JAK–STAT signaling pathway. (**a**) The expression levels of a number of key components of the JAK–STAT signaling pathway were evaluated by western blotting. The levels of p-Tyk2, p-JAK1, p-STAT1, and p-STAT2 were significantly reduced after HBV infection (Control *versus* HBV: *P<0.05, **P<0.01). However, the levels of Tyk2, JAK1, STAT1, and STAT2 were not significantly affected by HBV infection. The levels of p-Tyk2, p-JAK1, p-STAT1, and p-STAT2 were significantly reduced in HepG2 shTMEM2 cells than compared with HepG2 GFP cells (HepG2 GFP *versus* HepG2 shTMEM2: ^#^*P*<0.05, ^##^*P*<0.01). The levels of p-Tyk2, p-JAK1, p-STAT1, and p-STAT2 were significantly elevated in HepG2 TMEM2 cells compared with HepG2 GFP cells (HepG2 GFP *versus* HepG2 TMEM2: ^&^*P*<0.05). The levels of p-Tyk2, p-JAK1, p-STAT1, and p-STAT2 were significantly elevated in HepG2.2.15 TMEM2 cells compared with HepG2.2.15 GFP cells (HepG2.2.15 GFP *versus* HepG2.2.15 TMEM2: ^$^*P*<0.05, ^$$^*P*<0.05). No significant changes in cellular levels of total IRF9 were observed by HBV infection in any groups based on western blot analysis. HBsAg was significantly induced after HBV infection (Control *versus* HBV: *P<0.05, **P<0.01). (**b**) qPCR detection of the expression of MxA and OAS1 in HepG2 GFP and HepG2.2.15 cells. (*P<0.05 compared with HepG2 GFP cells without HBV infection; ^$^*P<*0.05 compared with HepG2.2.15 GFP cells without HBV infection; ^^^*P<*0.05 compared with HepG2.2.15 GFP cells infected with HBV.) Error bars are presented as S.D.

To investigate the molecular mechanism underlying the inhibitory effects of TMEM2 on HBV infection in HepG2 and HepG2.2.15 cells, western blot was used to evaluate the expression of a number of key components of the JAK–STAT signaling pathway in HBV-infected HepG2 and HepG2.2.15 cells. We found that the levels of p-Tyk2, p-JAK1, p-STAT1, and p-STAT2 in all HepG2 and HepG2.2.15 cells were reduced by HBV infection. No significant difference in the expression of Tyk2, JAK1, STAT1, and STAT2 was detected among any of the HBV-infected cells (Figure 4a). After HBV infection, the levels of p-Tyk2, p-JAK1, p-STAT1, and p-STAT2 in HepG2 shTMEM2 cells were lower than those in HepG2 cells, and the levels of p-Tyk2, p-JAK1, p-STAT1, and p-STAT2 in HepG2 TMEM2 cells were higher than those in HepG2 cells (Figure 4a). In addition, the levels of p-Tyk2, p-JAK1, p-STAT1, and p-STAT2 in HepG2.2.15 TMEM2 cells were higher than those in HepG2.2.15 cells following HBV infection (Figure 4a). After TMEM2 overexpression or knockdown, there were no significant differences in the cellular level of total interferon regulatory factor 9 (IRF9) in HepG2 and HepG2.2.15 cells (Figure 4a).

We also compared the expression of myxovirus resistance protein A (MxA) and 2′,5′-oligoadenylate synthetase 1 (OAS1), two interferon-inducible proteins in the downstream JAK–STAT signaling pathway, between HepG2 and HepG2.2.15 cells with and without HBV infection using RT-qPCR. We found that HBV infection significantly reduced the expression of MxA in all cells (Figure 4b). However, in the two groups of cells without HBV infection, the expression of MxA in HepG2 shTMEM2 cells was significantly lower than that in HepG2 cells (Figure 4b, *P<0.01). The expression of MxA in HepG2 TMEM2 cells was significantly higher than that in HepG2 cells (Figures 4b, *P*<0.01). In addition, overexpression of TMEM2 increased MxA expression in HepG2.2.15 cells irrespective of the presence of HBV infection. A similar expression profile was also obtained for OAS1. HBV infection significantly reduced the expression of OAS1 in HepG2 and HepG2 TMEM2 cells (Figure 4b). The expression of OAS1 in HepG2 shTMEM2 cells was significantly lower than that in HepG2 cells (Figure 4b, *P*<0.01). The expression of OAS1 in HepG2 TMEM2 cells was significantly higher than that in HepG2 cells (Figure 4b, *P*<0.05). The expression of OAS1 in HepG2.2.15 TMEM2 cells with or without HBV infection was significantly higher than that in HepG2.2.15 cells (Figure 4b, *P*<0.01).

### TMEM2 increased the translocation of IRF9 into nuclei

**Figure 5:**
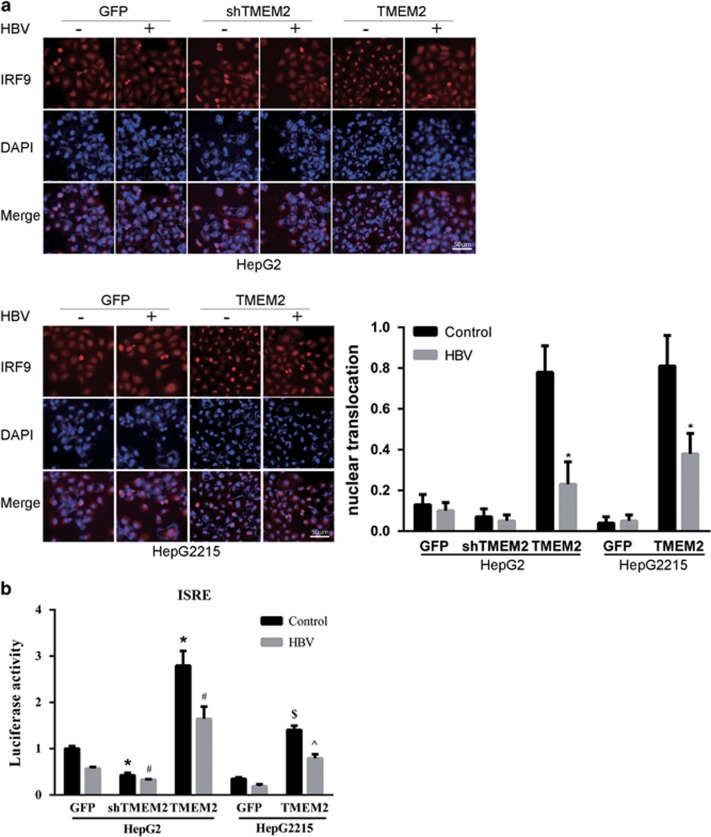
Effects of TMEM2 on the translocation of IRF9 into nuclei and the expression of MxA and OAS1. (**a**) IRF9 localization was analyzed by IFA. The level of IRF9 in the nuclei of HepG2 shTMEM2 cells did not change significantly in response to HBV infection compared with that in HepG2 GFP cells. In contrast, the level of IRF9 in the nuclei of non-HBV-infected HepG2 TMEM2 cells was remarkably higher than that in non-HBV-infected HepG2 GFP and HepG2 shTMEM2 cells. HBV infection reduced the level of IRF9 in the nuclei in HepG2 TMEM2 cells. Similar results were observed in HepG2.2.15 cells, which overexpressed TMEM2; nuclear translocation is represented by column shown on the right side of Figure 5a. (**b**) Using a plasmid that expresses a reporter gene (firefly luciferase) under the control of a promoter containing ISRE motifs, we showed that transfection of this plasmid into HepG2 TMEM2 cells, either with HBV infection or without, significantly increased luciferase activity compared with HepG2 GFP and HepG2 shTMEM2 cells (*P<0.05 compared with HepG2 GFP cells without HBV infection; ^#^*P*<0.05 compared with HepG2 GFP cells with HBV infection). Similar results were observed in TMEM2-overexpressing HepG2.2.15 cells (^$^*P*<0.05 compared with HepG2.2.15 GFP cells without HBV infection, ^^^*P*<0.05 compared with HepG2.2.15 GFP cells with HBV infection). When TMEM2 was silenced in HepG2 cells, the luciferase activity was markedly reduced. Error bars are presented as S.D.

To investigate whether TMEM2 could affect IRF9 translocation, immunofluorescence assay (IFA) was performed to detect IRF9 expression in both HepG2 and HepG2.2.15 cells. We found that TMEM2 overexpression enhanced IRF9 translocation into nuclei (Figure 5a), which was further confirmed using the luciferase reporter assay (Figure 5b). HepG2 TMEM2 cells transfected with the luciferase plasmid carrying interferon-stimulated response element (ISRE) motifs in the promoter region exhibited a significantly higher level of luciferase activity compared with that in HepG2 GFP cells transfected with the plasmid (Figure 5b). Similar results were observed in HepG2.2.15 TMEM2 cells. Whereas no significant decrease in the expression of IRF9 was observed in TMEM2 cells compared with the control cells based on IFA, a lower level of luciferase activity was observed in shTMEM2 cells (Figures 5a and b).

### IFN promoted the inhibitory effects of TMEM2 on HBV infection

**Figure 6:**
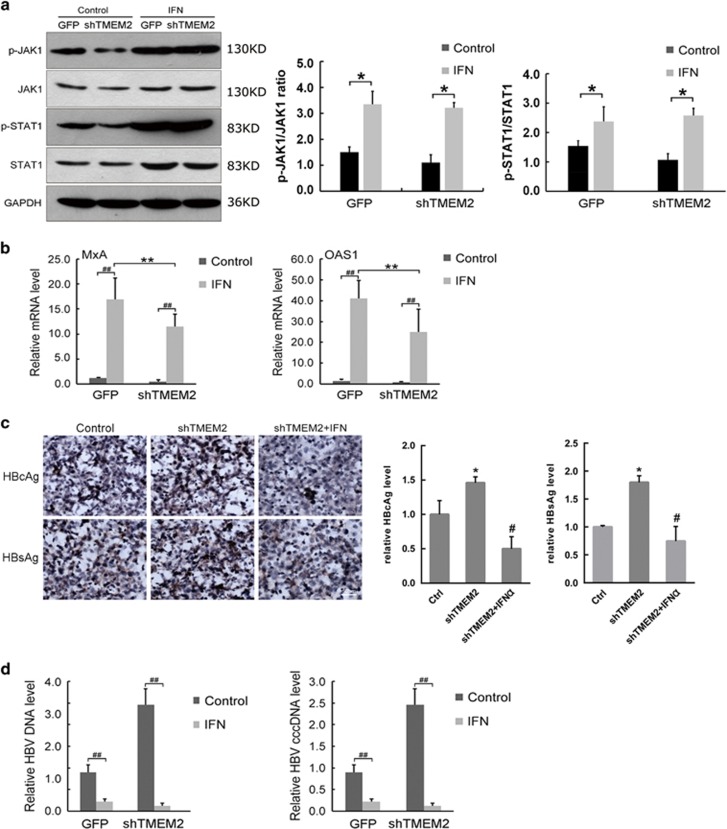
INF promoted the inhibitory effects of TMEM2 on HBV infection. (**a**) IFN pre-treatment increased the levels of p-JAK1 and p-STAT1 in HepG2 GFP and HepG2 shTMEM2 cells (Control *versus* IFN: *P<0.05). (**b**) IFN pre-treatment significantly increased the expression of MxA and OAS1 in HepG2 GFP and HepG2 shTMEM2 cells (^##^*P*<0.01). The expression of MxA and OAS1 in HepG2 shTMEM2 cells was significantly lower than that in HepG2 GFP cells (**P<0.01). (**c**) IFN pre-treatment reduced the levels of HBsAg and HBcAg in HepG2 shTMEM2 cells infected with HBV; the relative HBcAg and HBsAg levels are represented in the column graph on the right of Figure 6c. (**d**) IFN pre-treatment significantly reduced the mRNA levels of HBV DNA and HBV cccDNA in HepG2 GFP and HepG2 shTMEM2 cells infected with HBV (^##^*P*<0.01). Ctr, Control (HepG2 GFP). Error bars are presented as S.D.

To confirm the effects of TMEM2 on the JAK–STAT signaling pathway, HepG2 and HepG2 shTMEM2 cells were infected with HBV after pre-treatment with interferon (IFN), and the expression of a number of key components of the JAK–STAT signaling pathway was evaluated by western blot and RT-qPCR analyses. The western blot results demonstrated that the levels of p-JAK1 and p-STAT1 were significantly increased in HepG2 and HepG2 shTMEM2 cells in response to IFN pre-treatment (Figure 6a). The RT-qPCR assay results showed that IFN pre-treatment significantly increased the expression of MxA and OAS1 in HepG2 and HepG2 shTMEM2 cells (Figure 6b, *P*<0.01). However, the levels of MxA and OAS1 in HepG2 shTMEM2 cells were significantly lower than those in HepG2 cells (Figure 6b, *P*<0.01). In addition, IFN pre-treatment reduced the levels of HBsAg and HBcAg in HepG2 shTMEM2 cells after HBV infection based on IHC analysis (Figure 6c). The RT-qPCR results demonstrated that IFN pre-treatment significantly reduced the levels of HBV DNA and HBV cccDNA in HepG2 and HepG2 shTMEM2 cells (Figure 6d, *P*<0.01).

### JAK1 inhibitor repressed the inhibitory effects of TMEM2 on HBV infection

To further evaluate the effects of TMEM2 on the JAK–STAT signaling pathway, HepG2.2.15 and HepG2.2.15 TMEM2 cells were pre-treated with a JAK1 inhibitor and infected with HBV. The expression of a number of key components of the JAK–STAT signaling pathway, the levels of HBsAg and HBcAg, and the levels of HBV DNA and HBV cccDNA were evaluated by western blotting, IHC, and RT-qPCR, respectively.

**Figure 7:**
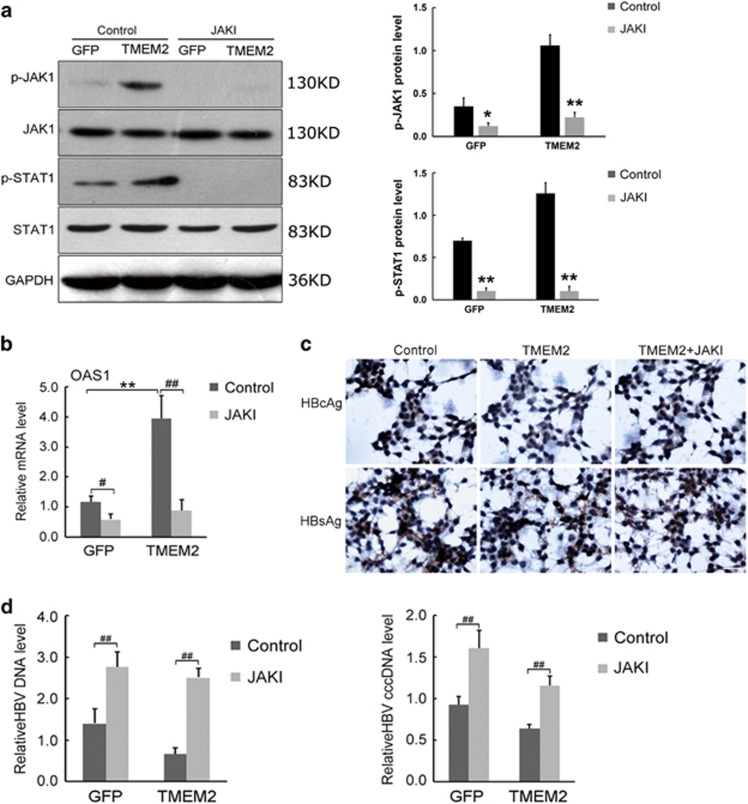
JAK1 inhibitor repressed the inhibitory effects of TMEM2 on HBV infection. (**a**) JAK1 inhibitor significantly reduced the levels of p-JAK1 and p-STAT1 in HepG2.2.15 GFP and HepG2.2.15 TMEM2 cells. However, the expression of JAK1 and STAT1 in HepG2.2.15 GFP and HepG2.2.15 TMEM2 cells was not significantly affected by JAK1 inhibitor; quantitation of p-JAK1 and p-STAT1 is shown in the column graph on the right of Figure 7a. (**b**) JAK1 inhibitor significantly reduced the expression of OAS1 in HepG2.2.15 GFP and HepG2.2.15 TMEM2 cells (^#^*P*<0.05, ^##^*P*<0.01). Prior to JAK1 inhibitor pre-treatment, the expression of OAS1 in HepG2.2.15 TMEM2 cells was significantly higher than that in HepG2.2.15 GFP cells (**P<0.01). (**c**) JAK1 inhibitor increased the levels of HBsAg and HBcAg in HepG2.2.15 TMEM2 cells infected with HBV. (**d**) JAK1 inhibitor significantly increased the levels of HBV DNA and HBV cccDNA in HepG2.2.15 GFP and HepG2.2.15 TMEM2 cells infected with HBV (^##^*P*<0.01). Ctr, Control (HepG2.2.15 GFP). Error bars are presented as S.D.

The western blot results revealed that the levels of p-JAK1 and p-STAT1 in HepG2.2.15 and HepG2.2.15 TMEM2 cells were significantly reduced by JAK1 inhibitor. However, there were no significant changes in the expression of JAK1 and STAT1 in HepG2.2.15 and HepG2.2.15 TMEM2 cells after pre-treatment with JAK1 inhibitor (Figure 7a). RT-qPCR revealed significant changes in OAS1 expression in HepG2.2.15 and HepG2.2.15 TMEM2 cells after pre-treatment with JAK1 inhibitor (Figure 7b, *P*<0.01). In addition, the expression of OAS1 in HepG2.2.15 TMEM2 cells without JAK1 inhibitor pre-treatment was significantly higher than that in HepG2.2.15 cells (Figure 7b, *P*<0.01). The IHC results demonstrated that the levels of HBsAg and HBcAg in HepG2.2.15 TMEM2 cells were increased by pre-treatment with JAK1 inhibitor after HBV infection (Figure 7c). The RT-qPCR results demonstrated that JAK1 inhibitor significantly increased the levels of HBV DNA and HBV cccDNA in HepG2.2.15 and HepG2.2.15 TMEM2 cells (Figure 7d, *P*<0.01).

TMEM2 is a type II transmembrane protein. The biological function of TMEM2 is not yet fully understood. The association of TMEM2 mutations with human deafness was first reported in 2000.^7^ Animal experiments have also shown that TMEM2 is involved in cardiac development in zebrafish.^8, 9^ Expression analyses suggest that TMEM2 is widely expressed in various human tissues, especially liver tissue (http://www.proteinatlas.org/ENSG00000135048-TMEM2/tissue). However, whether TMEM2 is involved in the development of liver diseases is not clear. Therefore, in the present study, we investigated the role of TMEM2 in the pathogenesis of HBV infection in HepG2 and HepG2.2.15 cells.

First, we confirmed that TMEM2 expression in normal liver tissues and HepG2 cells was significantly higher than that in the liver tissues of patients with HBV infection and HepG2.2.15 cells with HBV genomic DNA, respectively. The results suggested that TMEM2 was negatively associated with HBV infection in HepG2 and HepG2.2.15 cells. In addition, we found that, after HBV infection, the levels of HBsAg, HBcAg, HBV DNA, and HBV cccDNA in HepG2 TMEM2 and HepG2.2.15 TMEM2 cells, in which TMEM2 was overexpressed, were significantly lower than those in HepG2 and HepG2.2.15 cells, respectively. In addition, the levels of HBsAg, HBcAg, HBV DNA, and HBV cccDNA in HepG2 shTMEM2 cells, in which the expression of TMEM2 was silenced, were significantly higher than those in HepG2 cells after HBV infection. These results suggested that TMEM2 inhibited HBV infection in HepG2 and HepG2.2.15 cells.

**Figure 8:**
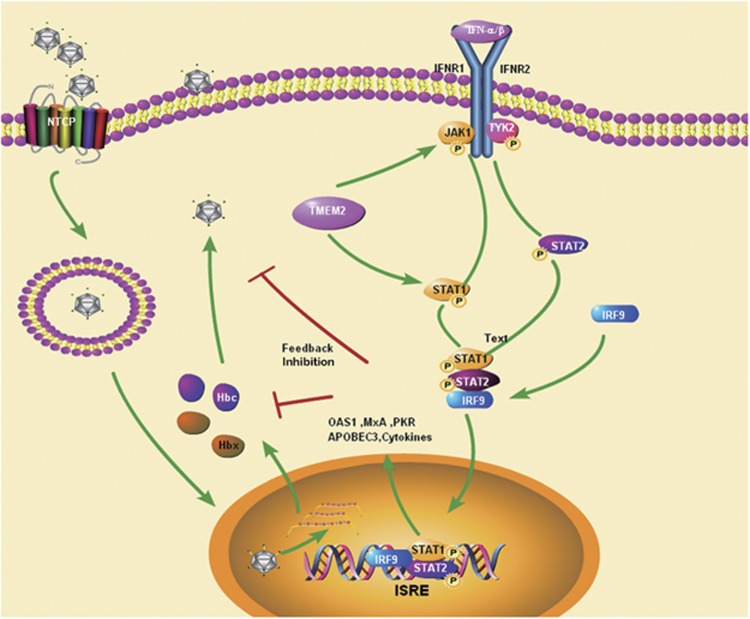
A hypothetical model of TMEM2 exerting its antiviral functions. TMEM2 activates the JAK–STAT signaling pathway and regulates the levels of p-Tyk2, p-JAK1, p-STAT1, p-STAT2, MxA and OAS1 in HepG2 and HepG2.2.15 cells. IFN can also activate the JAK–STAT signaling pathway and upregulate TMEM2 expression, leading to enhanced inhibitory effects of IFN on HBV production. Furthermore, TMEM2 increases the translocation of IRF9 into nuclei, thus enhancing the expression of a luciferase reporter gene under the control of ISRE motifs

Previous studies have shown that multiple signaling pathways, such as NF-κB, Smad7-TGF-β, REB/PKA, and JAK–STAT, are involved in the pathogenesis of HBV infection.^6, 10, 11, 12^ Among these signaling pathways, the JAK–STAT pathway is involved in the anti-HBV activity of IFN. In addition, HBV and HBV antigens can regulate the expression of JAK–STAT signaling pathway molecules and antiviral proteins in hepatocellular models *in vitro*. Furthermore, the reduced STAT1 methylation and subsequent increased STAT1–PIAS1 interaction contributed to the antagonistic activity of IFN-alpha against HBV.^6^ Therefore, we examined whether the JAK–STAT signaling pathway was involved in the inhibitory effect of TMEM2 on HBV infection. First, we investigated whether TMEM2 could regulate the expression of the JAK–STAT signaling pathway. Our results showed that TMEM2 overexpression activated the JAK–STAT signaling pathway and TMEM2 knockdown decreased the levels of p-Tyk2, p-JAK1, p-STAT1, and p-STAT2 in HepG2 and HepG2.2.15 cells, suggesting that TMEM2 regulates the JAK–STAT signaling pathway. This finding was further supported by the expression levels of MxA and OAS1, two downstream molecules in the JAK–STAT signaling pathway. IFN has been shown to activate the JAK–STAT signaling pathway. We observed that IFN treatment increased the expression of MxA and OAS1 in HepG2 and HepG2.2.15 cells, and this effect was compromised by reduced TMEM2 expression, leading to attenuated inhibitory effects of IFN on HBV production. Furthermore, we explored whether blocking the JAK–STAT signaling pathway could abolish the anti-HBV effects of TMEM2. Our data revealed that pre-treatment with JAK1 inhibitor resulted in elevated levels of HBsAg, HBcAg, HBV DNA, and HBV cccDNA in TMEM2-overexpressing HepG2 and HepG2.2.15 cells. Interestingly, as shown in Figure 8, TMEM2 caused an increase in the translocation of IRF9 into nuclei, but no effect on IRF9 expression was detected. The increased levels of nuclear IRF9 enhanced the expression of a luciferase reporter gene under the control of ISRE motifs. Given that TMEM2 increased the phosphorylation of STAT1 and STAT2, and promoted the translocation of IRF9 into nuclei, it would be interesting to determine whether TMEM2 is involved in the formation of the ISGF3 complex, a key player in type 1 interferon signaling. Interferon regulatory factors (IRFs) are a family of transcription factors that mediate the transcriptional regulation of interferon genes and IFN-inducible genes.^13^ IRF9 is an immunomodulatory transcription factor that is important for the response to IFN-α downstream of the JAK/STAT signaling pathway.^14, 15^

Taken together, our results suggest that TMEM2 inhibits HBV infection in HepG2 and HepG2.2.15 by activating the JAK–STAT signaling pathway.

Bioinformatics analyses have shown that TMEM2 contains a short intracellular region and a long extracellular region that includes the G8 and GG domains. The G8 domain has also been identified in a number of proteins such as PKHD1 and KIAA1109, which are involved in certain human diseases. For example, it has been reported that the transmembrane protein PKHD1 is associated with kidney and liver diseases.^16^ Binding to ligands can phosphorylate the intracellular region of PKHD1, which then regulates a variety of cellular activities.^16, 17^ How TMEM2 regulates the JAK–STAT signaling pathway, that is, promotes the phosphorylation of p-Tyk2, p-JAK1, p-STAT1, and p-STAT2, was not investigated in the present study.

Current studies investigating HBV receptors have mainly focused on the sodium taurocholate cotransporting polypeptide (NTCP), which is the receptor of both HBV and HDV.^18, 19, 20, 21^ The interaction between the HBV preS1 domain and NTCP mediates the entry of HBV into cells. Our previous *in vivo* study confirmed that NTCP is the receptor of HBV and demonstrated that the NTCP mutation (p.Ser267Phe) was associated with a reduced prevalence of acute and chronic liver failure.^22^ In particular, we identified five cases of the p.Ser267Phe homozygous mutation among 1899 patients with chronic HBV infection. Given that five homozygous patients were infected with HBV, we speculate that there is more than one route of HBV cellular entry. Characterization of the biological functions of TMEM2 is useful to understand the mechanism of HBV infection. It is not clear whether TMEM2 is a HBV receptor involved in HBV infection through the G8 and GG domains. In addition, the role of TMEM2 in HBV infection should be investigated *in vivo*. Whether the structure of TMEM2 is altered during HBV infection and whether TMEM2 interacts with NTCP should be investigated in future investigations.

## Materials and Methods

### Tissue and cell cultures

This study was approved by the Ethics Committee of the Third Affiliated Hospital of Sun Yat-sen University for Human Study, and was conducted according to the principles of the Declaration of Helsinki (2013).

Diagnosis of chronic hepatitis B (CHB) infection was based on positive results for serological HBsAg and persistently or recurrently elevated levels of serum alanine aminotransferase for more than 6 months, according to the criteria of the Chinese Society of Hepatology and Chinese Society of Infectious Diseases and the Chinese Medical Association.^23^ Liver tissues were obtained by liver biopsy. Healthy and CHB liver tissues were formalin-fixed, paraffin-embedded and sectioned for immunohistochemistry assays, or immediately frozen for western blot and RT-qPCR assays.

HepG2.2.15 cells containing the complete HBV genome and capable of stable HBV expression and replication in culture, and a non-HBV-containing HepG2 cell line (ATCC, Manassas, VA, USA), were maintained in exponential growth phase in DMEM (Life Technologies, Carlsbad, CA, USA) supplemented with 10% fetal bovine serum, 100 units/ml penicillin, and 0.1% (w/v) streptomycin at 37 °C in a humidified atmosphere of 5% CO_2_. G418 (500 mg/l) was added to the HepG2.2.15 culture for selection.

### Establishment of stable TMEM2-overexpressing or silenced cell lines

Establishment of stable TMEM2-overexpressing or silenced cell lines was performed according to a previous study with slight modifications.^24^ To obtain lentiviral expression of TMEM2, TMEM2 cDNA with an in-frame stop codon was amplified by PCR and inserted into vector LV-003 with the CMV promoter (Forevergen Biosciences, Guangzhou, China). Vector LV-008 (Forevergen Biosciences, Guangzhou, China) with the U6 promoter was used to generate small hairpin RNAs. The following oligonucleotides were sub-cloned into the HpaI/XhoI sites: the small hairpin RNA of TMEM2 (shTMEM2) and the TMEM2 shRNA sequences: sense 5′-CACCGCTTGCCCTTTGGGTCCTATACGAATATAGGACCCAAAGGGCAAGC-3′ and antisense 5′-AAAAGCTTGCCCTTTGGGTCCTATATTCGTATAGGACCCAAAGGGCAAGC-3′. Lentiviral production was performed as previously described.^8^ Briefly, HEK293T cells were co-transfected with LV-003-TMEM2 (or LV-008-shTMEM2) and packaging vectors, and the supernatant was collected after 48 and 72 h of incubation. Lentiviruses were recovered after centrifugation at 25 000 r.p.m. for 1.5 h in a Beckman Instrument (Fullerton, CA, USA) and resuspended in PBS. The virus was then used to infect cells in the presence of polybrene (6 mg/ml). After 48 h, the cells were cultured in medium containing puromycin (2 *μ*g/ml) for 10–15 days to generate stable cell lines. Stable TMEM2-overexpressing or silenced cell lines were confirmed by western blot, RT-qPCR and the MTT assay.

### Immunohistochemistry

Immunohistochemistry was performed using formalin-fixed and paraffin-embedded healthy and CHB liver tissue sections or fixed cultured cells. The tissue sections or cells were incubated with the primary antibodies (TMEM2 antibody 1:100, Aviva Systems Biology, San Diego, CA, USA; anti-HBs 1:50, anti-HBc 1:50, BOSTER, Wuhan, China) at 4 °C overnight. Next, peroxidase-labeled secondary antibodies (Dako K5007, Carpinteria, CA, USA) were applied to the slides for 30 min at 37 °C, followed by development using diaminobenzidine. For the negative control, the primary antibody was replaced with PBS.

### HBV infection

HBV infection was performed as described previously.^25, 26, 27, 28^ Briefly, 10 ml of vein blood was collected from CHB patients at the Department of Infectious Disease between 2012 and 2014, centrifuged at 2000 × *g* for 30 min to obtain HBV serum, and subjected to the new Roche Cobas AmploPrep/Cobas TaqMan HBV test, version 2.0, real-time PCR assay to detect and quantify HBV DNA. Serum samples with a HBV DNA load exceeding 10e8 IU/ml were collected, and HepG2 and HepG2.2.15 cells were cultured in 6-well plates or on glass coverslips in a 24-well plate, pre-treated with 2% DMSO for 2–4 days, and then incubated in DMEM containing 2% DMSO and 10% HBV serum. After 48 h of incubation, the cells were washed five times with PBS and then subjected to western blot, RT-qPCR, and immunohistochemistry assays.

To evaluate the influence of IFN and JAK1 inhibitor on the inhibitory effects of TMEM2 on HBV infection, HepG2 shTMEM2 and HepG2.2.15 TMEM2 cells were treated with IFN (PeproTech, Rocky Hill, NJ, USA) and JAK1 inhibitor (Santa Cruz Biotechnology, CA, USA) for 48 h, respectively. Subsequently, they were harvested for western blot, RT-qPCR, and immunohistochemistry assays.

### HBV DNA and cccDNA quantification

HBV DNA and cccDNA quantification was performed as described previously.^25, 29^ HBV DNA was isolated according to standard genomic DNA isolation methods.^30^ Briefly, HBV-infected cells were lysed at 65 °C for 4 h in lysis buffer (50 mM Tris–HCl, pH 8.0, 50 mM EDTA, 100 mM NaCl, 1% SDS) supplemented with proteinase K (200 *μ*g/ml). HBV DNA was isolated from the lysed cells using the QIAGEN QIAamp DNA mini kit (Qiagen, Hilden, Germany). The DNA was quantified using specific primers, 5′-ATGGAGAACACAACATCAGG-3′ and 5′-GAGGCATAGCAGCAGGATG-3′, by real-time PCR. A total of 1000 ng of isolated HBV DNA was digested with 20–40 units of plasmid-safe DNase (Epicentre Technologies #E3101K, Madison, WI, USA) in a total volume of 20 *μ*l at 37 °C for 8 h. The digested HBV DNA was inactivated with DNase at 70 °C for 30 min. Two microliter of the 20-*μ*l reaction was then added to a 20-*μ*l real-time PCR reaction. Primers for cccDNA detection (5′-GTCTGTGCCTTCTCATCTGCC-3′ 5′-ACAGCTTGGAGGCTTGAACAG-3′) were validated and used for real-time PCR.

### Immunofluorescence assay

The IFA was performed to localize IRF9. The cells were fixed with 4% paraformaldehyde in PBS containing 0.5% Triton X-100 at 4 °C for 30 min. After three washes with PBS, anti-IRF9 antibody (1:100 dilution) was added to the cells and incubated at room temperature for 1 h. The cells were then washed with PBS and incubated with PE-conjugated secondary antibodies (Invitrogen, 1:1000 dilution). Subsequently, the cells were washed with PBS and mounted using VectaShield with DAPI. IRF9 staining was examined using fluorescence microscopy.

### Luciferase reporter assay

A luciferase reporter assay was performed to assess the effects of TMEM2-induced IRF9 translocation into nuclei. Plasmid pISRE-TA-Luc (Clontech) containing five copies of consensus ISRE upstream of the firefly luciferase gene was used for cell transfection. Plasmid pTA-Luc (Clontech) lacking the enhancer element was used for background determination. Plasmid pRL-CMV (Promega) expressing the Renilla luciferase protein was used to normalize the transfection efficiency. To measure IRF9-induced ISRE activities, 5 × 10^4^ cells per well were seeded in a 24-well plate 1 day before transfection. Five hundred nanograms of pISRE-TA-Luc or pTA-Luc with 1 ng of pRL-CMV were used for cell transfection with 2 *μ*l of Lipofectamine 2000 according to the manufacturer's instructions. Forty-eight hours after transfection, dual luciferase assays were performed using the cell lysates according to established protocols.

### Real-time quantitative PCR

Total RNA was isolated from tissue samples using TRIzol reagent (Invitrogen, Carlsbad, CA, USA) according to the manufacturer's instructions. RNA was reverse transcribed using the ReverTra Ace qPCR RT Kit (Toyobo Biochemicals, Osaka, Japan) according to the manufacturer's protocol. The mRNA of the housekeeping gene glyceraldehyde-3-phosphate dehydrogenase (GAPDH) served as an endogenous standard. The primers used for RT-qPCR assay were as follows: TMEM2 forward, 5′-GGAGATATGCTCCGTCTGACC-3′ and TMEM reverse, 5′-CATCTGACTTGCCATACAAGGT-3′ MxA forward, 5′-GTTTCCGAAGTGGACATCGCA-3′ and MxA reverse, 5′-CTGCACAGGTTGTTCTCAGC-3′ OAS1 forward, 5′-CGTGTTTCCGCATGCAAAT-3′ and reverse, 5′-ACCTCGGAAGCACCTTTCCT-3′ GAPDH forward, 5′-GAGTCAACGGATTTGGTCGT-3′ and reverse 5′-GACAAGCTTCCCGTTCTCAG-3′. All PCR reactions were performed using a SYBR Green Master kit (Roche, Basel, Switzerland) according to the manufacturer's protocol. The threshold cycle (C_t_) value of each sample was calculated, and the relative mRNA level was normalized to the GAPDH mRNA value.

### Western blot analysis

Total protein was isolated from cells or tissue samples using RIPA lysis buffer. The protein concentration was determined using the Bradford assay. Equal amounts of total protein were separated by SDS-PAGE electrophoresis, transferred to PVDF membranes, and blocked with 5% skim milk powder at room temperature for 1 h. Next, the PVDF membranes were washed with TBST containing NaCl, Tris-HCl, and Tween-20 and incubated with primary antibodies against target proteins at 4 °C overnight, followed by two washes with TBST. TMEM2 antibody was purchased from Aviva Systems Biology. Antibodies against p-Tyk2, Tyk2, p-JAK, JAK, p-STAT1, STAT1, p-STAT2, STAT2, IRF9, and GAPDH were obtained from Cell Signaling Technology. PVDF membranes were incubated with the appropriate secondary antibodies at room temperature for 1 h and washed three times with TBST. Protein bands were visualized by chemiluminescence (ECL, Forevergen, Guangzhou, China).

### Statistical analyses

Data from all cell culture-based assays are presented as the mean±S.D. ANOVA tests were used to evaluate differences among groups. A *P-*value <0.05 was considered statistically significant.

## Abbreviations

HBV: hepatitis B virus
CHB: chronic hepatitis B
HBsAg: hepatitis B virus surface antigen
HBcAg: hepatitis B virus core antigen
MxA: myxovirus resistance protein 1
OAS1: 2', 5'-oligoadenylate synthetase 1
IFN: interferon
NTCP: sodium taurocholate cotransporting polypeptide
