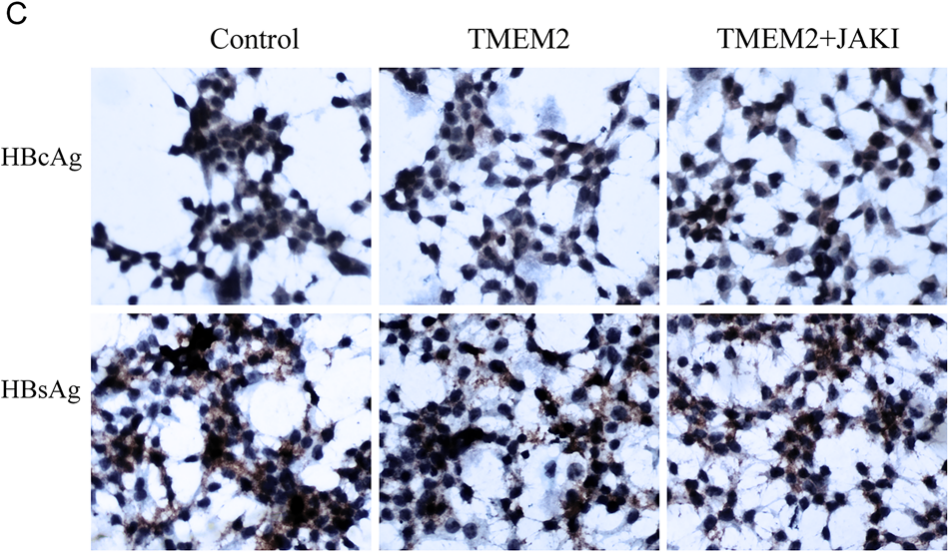# Correction: TMEM2 inhibits hepatitis B virus infection in HepG2 and HepG2. 2. 15 cells by activating the JAK–STAT signaling pathway

**DOI:** 10.1038/s41419-025-07706-w

**Published:** 2025-06-12

**Authors:** X. Zhu, C. Xie, Y. -M Li, Z. -L Huang, Q. -Y Zhao, Z. -X Hu, P. -P Wang, Y. -R Gu, Z. -L Gao, L. Peng

**Affiliations:** 1 Department of Infectious Diseases, Third Affiliated Hospital of Sun Yat-sen University, 600# Tianhe Road, Guangzhou, Guangdong Province China; 2 Guangdong Provincial Key Laboratory of Liver Diseases, Third Affiliated Hospital of Sun Yat-sen University, Guangzhou, Guangdong Province China; 3 Key Laboratory of Tropical Disease Control, Ministry of Education, Sun Yat-sen University, Guangzhou, Guangdong Province China; 4 Department of Traditional Chinese Medicine, Third Affiliated Hospital of Sun Yat-sen University, 600# Tianhe Road, Guangzhou, Guangdong Province China

Correction to: *Cell Death & Disease* 10.1038/cddis.2016.146, published online 2 June 2016

Due to an oversight in figure selection, the original representative image of Figure 7C (HBcAg Control, HBcAg TMEM2+JAKI) is incorrect. However, this does not affect the experimental conclusions. Therefore, it is necessary to correct Figure 7C (HBcAg Control, HBcAg TMEM2+JAKI).